# Determination of Glyphosate, Glufosinate, and Their Major Metabolites in Tea Infusions by Dual-Channel Capillary Electrophoresis following Solid-Phase Extraction

**DOI:** 10.1155/2022/5687025

**Published:** 2022-04-01

**Authors:** Manh Huy Nguyen, Thanh Dam Nguyen, Minh Tuan Vu, Hong Anh Duong, Hung Viet Pham

**Affiliations:** ^1^Key Laboratory of Analytical Technology for Environmental Quality and Food Safety Control (KLATEFOS), VNU University of Science (VNU-HUS), Vietnam National University, Hanoi (VNU), 334 Nguyen Trai Street, Thanh Xuan District, Hanoi, Vietnam; ^2^Research Centre for Environmental Technology and Sustainable Development (CETASD), VNU University of Science (VNU-HUS), Vietnam National University, Hanoi (VNU), 334 Nguyen Trai Street, Thanh Xuan District, Hanoi, Vietnam

## Abstract

In this study, two analytical procedures were developed and validated using dual-channel capillary electrophoresis-coupled contactless conductivity detection (CE-C4D) followed by solid-phase extraction (SPE) for simultaneous determination of glyphosate (GLYP), glufosinate (GLUF), and their two major metabolites, aminomethylphosphonic acid (AMPA) and 3-(methylphosphinico) propionic acid (MPPA), respectively, in a popular beverage such as tea infusions. GLYP, GLUF, and AMPA were analyzed in the first channel using background electrolyte (BGE) of 1 mM histidine (His) adjusted to pH 2.75 by acetic acid (Ace). In contrast, MPPA was quantified in the second channel with a BGE of 30 mM His adjusted to pH 6.7 by 3-(N-morpholino) propanesulfonic acid (MOPS) and 10 µM of cetyltrimethylammonium bromide (CTAB). In addition, the samples of tea infusions were treated using SPE with 10 mL of 0.5 mM HCl in methanol as eluent. At the optimized conditions, the method detection limit (MDL) of GLYP, GLUF, AMPA, and MPPA is 0.80, 1.56, 0.56, and 0.54 *μ*g/l, respectively. The methods were then applied to analyze four target compounds in 16 samples of tea infusions. GLYP was found in two infusion samples of oolong tea with concentrations ranging from 5.34 to 10.74 µg/L, and GLUF was recognized in three samples of green tea infusion in the range of 45.1–53.9 µg/L.

## 1. Introduction

Since its commercialization by Monsanto Company (USA) under the name Roundup in 1974, glyphosate (GLYP) has become the world's most commonly used herbicide for broadleaf weed control and as a plant growth regulator [1]. Besides GLYP, glufosinate (GLUF), a similar phosphorus herbicide, is also widely used because of its broad spectrum of action, including against GLYP-resistant weeds [2]. On the other hand, according to the World Health Organization (WHO), glyphosate has potential carcinogenic effects [3]. Furthermore, acute glufosinate exposure can cause toxicities for the central nervous and respiratory systems [4].

Given the long history of use and the enormous consumption volume, the residues of GLYP and GLUF were found in soil [1, 5], environmental water [6–9], plant [10, 11], food [12, 13], and also in human [14–16]. This increases the risk of human exposure to these herbicides and raises concerns about human health and environmental hazards. As a result, the European Union has stringent regulations regarding the existence of pesticides in the water meant for human consumption, such as a maximum concentration of 0.1 µg/L for each pesticide and a total concentration of less than 0.5 µg/L [17].

Tea is one of the most popular beverages in the world after water, and it is brewed by steeping tea leaves/buds in hot/boiling water to make tea infusions. The tea is cultivated in old orchards, which provide an ideal setting for weed development [18], which is progressively controlled by herbicides like GLYP or GLUF. Consequently, there is increasing concern that tea infusions may be a source of herbicide residues' exposure to humans. Therefore, it is essential to determine the concentration of GLYP and GLUF and their primary metabolites aminomethylphosphonic acid (AMPA) and 3-(methylphosphinico)propionic acid (MPPA) in tea infusions [19].

It is challenging to simultaneously analyze GLYP, GLUF, and their metabolites in aqueous matrices due to their characteristics of highly polar, lack of chromogenic and fluorescent groups. The methods of liquid chromatography (LC) [20–26], high-performance liquid chromatography (HPLC) [19], and ultra-high performance liquid chromatography (UHPLC) [27–30] coupled to tandem mass spectrometry (MS/MS) are commonly used methods, although these methods usually need the derivatization. On the other hand, the derivatization step can be omitted when ion chromatography coupled to tandem mass spectrometry (IC-MS/MS) is used [31]. However, using LC, GC, or IC-MS/MS increases analytical costs and requires skilled technicians. In contrast, capillary electrophoresis using contactless conductivity detection (CE-C^4^D) is likely the more appropriate method with its advantages of simple and low cost [32].

Although some research [33–38] has used the CE technique to determine GLYP residues with GLUF, AMPA, and MPPA in environmental and drinking water samples, the use of C^4^D detection is few and lacks updates. Hong Heng See et al. used the CE-C^4^D method and electrokinetic injection technique to analyze those substances in drinking water with a linear range of 0.01–0.1 µM [39, 40]. However, the analytical procedures in these works have only been illustrated for the relatively simple matrix of tap water and are difficult to apply to more complex samples. Another study used the software to determine thermodynamic acidity constants and limit ionic mobilities of glyphosate and its metabolites and application in the separation of glyphosate [41]. Regardless, GLUF and MPPA were not the targets of that work. Therefore, to fill the gap, this study aimed to develop the application of the dual-channel CE-C^4^D combined with solid-phase extraction (SPE) to simultaneously determine GLYP, GLUF, AMPA, and MPPA in tea infusions. A new approach using a two-channel CE system to simultaneously analyze these compounds is in only one run. In addition, by analyzing on two different channels, the process of optimization was simpler.

## 2. Materials and Methods

### 2.1. Chemicals and Apparatus

All chemicals used in this study were of reagent grade. GLYP (99.1%), GLUF (99.5%), AMPA (99.7%), and MPPA (99.5%) were purchased from CPAchem (Bogomilovo, Bulgaria), and each stock solution was prepared in methanol at a concentration of 1,000 µg/mL. These stock solutions were then used for the preparation of standard solutions by diluting with deionized water, which was purified with a Simplicity UV system—Millipore (USA). Histidine (His), 3-(N-morpholino)propanesulfonic acid (MOPS), cetyltrimethylammonium bromide (CTAB), formic acid (For), citric acid (Cit), acetic acid (Ace), lactic acid (Lac), and succinic acid (Suc) were used to prepare the background electrolyte (BGE) solutions, and these chemicals were bought from Sigma-Aldrich (Darmstadt, Germany) or Merck KGaA (Darmstadt, Germany) except lactic acid from Xilong Chemical (Guangdong, China). Besides, methanol (MeOH), hydrochloric acid (HCl), and sodium hydroxide (NaOH) were used in SPE procedures. The purities of these chemicals were greater than 99%, except lactic acid (90%).

A purpose-made CE instrument with two channels independently working was used for all experiments. The setup and operation of the CE system were demonstrated in previous publications [42–44]. The capillaries used in this system were fused silica obtained from BGB Analytik AG (Böckten, Switzerland) with 50 µm ID and 365 µm OD. Before use, the capillaries were preconditioned with 0.1 M NaOH for 10 min and deionized water for 10 min prior to flushing with the BGE solutions.

The SPE system from Supelco (USA) was used for SPE producers using six mL Oasis WAX cartridges (containing 500 mg sorbents). In addition, a vacuum evaporator system of BUCHI (Switzerland) and MGS-2200 nitrogen sample concentrator (Eyela, Japan) were used to concentrate the SPE extract.

### 2.2. Sample Collection and Sample Treatment

Sixteen samples of tea products were collected from the typical supermarkets in Hanoi in the last quarter of 2021. The sample preparation was adapted from Phan Thi, LA. et al. (2020) [45], and in particular, 1.0 gram of dry tea leaves of each sample was weighed and transferred to a glass flask, and then, 100 mL boiled water was added. The flask was kept for 10 min, and then, the aqueous solution was filtered through a PTFE filter with 0.45 µm size.

An Oasis WAX cartridge was preconditioned with 5 mL of MeOH followed by 5 mL of deionized water. Then, 100 mL of tea infusion sample was loaded through at a 1 mL/min flow rate. Next, the SPE cartridge was washed with 5 mL of deionized water and 5 mL of methanol and then dried for 10 min. Next, the target compounds in the cartridge were eluted with 10 mL of 0.5 M HCl in MeOH at a rate of 0.5 mL/min. Finally, the extract was dried under a gentle nitrogen stream at 60 C, and the obtained solid residue was dissolved in 1 mL of deionized water. The final solution was filtered through a 0.2 *μ*m nylon membrane filter before CE-C^4^D analysis.

### 2.3. Analytical Procedures

In this study, GLYP, GLUF, and AMPA were simultaneously determined on the first channel of the dual CE-C^4^D system using the BGE composed of 1 mM His adjusted to pH 2.75 by Ace and a voltage of +20 kV from the detection end of the capillary. The second channel was used to analyze MPPA with the optimized BGE of 30 mM His/MOPS (pH = 6.7) and then added 10 µM of CTAB and an applied voltage of +20 kV. Both capillaries of the two channels have the exact total and effective lengths of 70 and 62 cm, respectively.

## 3. Results and Discussion

### 3.1. Development of the CE-C4D Methodology for Determination of GLYP, GLUF, and MPPA

**Figure 1:**
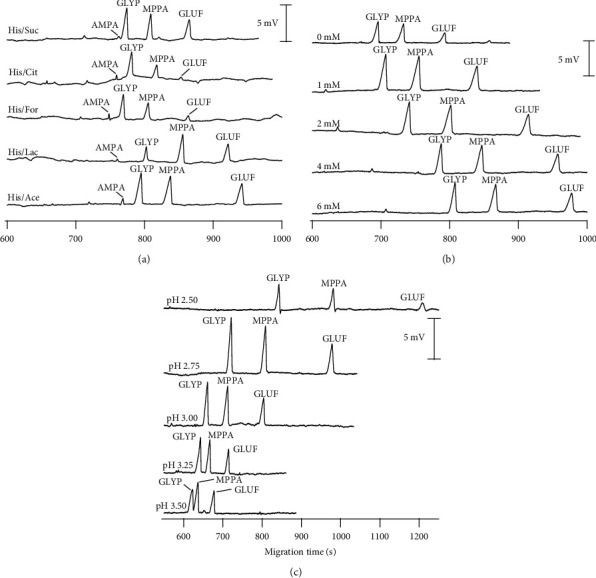
Electropherogram for the optimization of BGE compositions for determination of GLYP, GLUF, and MPPA. CE conditions: voltage: 20 kV from the detection side; uncoated fused silica capillary with ID = 50 µm, Lt = 70 cm, and Leff = 62 cm.

In the selection of BGE, the primary consideration is the ionization characteristic of the analytes. Based on the acid-base property of the four target substances, GLYP, GLUF, and AMPA can exist as the cation (pH < pK_a1_, the –NH_2_ group is protonated to becomes –NH_3_^+^) or anion (pH > pK_a2_, two acidic O–H groups are deprotonated), while MPPA can only be analyzed as anion (pH > pK_a_) due to the lack of an amino group. Besides, the BGEs composed of low His concentrations at acidic pHs have been shown to be good separation efficient of phosphate ions [46]. Accordingly, BGEs composed of 4 mM His adjusted to pH 3.0 by different commonly used acids (Ace, Cit, For, Lac, and Suc) were investigated (Figure 1).

As shown in Figure 1(a), AMPA signals in all conditions were minimal because this compound mainly existed in the form of a zwitterion at pH 3.0, resulting in a total charge of approximately zero. Therefore, AMPA should be surveyed with the different BGE conditions at a higher pH on another channel while three remaining analytes were separated in one capillary. The BGE with the composition of His/Ace produced a stable baseline, higher peaks, and better separation for GLYP, GLUF, and MPPA, and as a significant result, this buffer was chosen for further modification of BGE concentrations and pH, as it was previously proven that the signals in CE-C^4^D may also be dependent on these parameters.

Figure 1(b) illustrates the effect of various His concentrations ranging from 0 to 6 mM on the separation performance of GLYP, GLUF, and MPPA. As it can be seen, an increase in BGE concentration was associated with a more extended separation time due to the increase in the velocity of solutions at higher concentrations. In His absence, the acquired signals were markedly reduced, possibly due to a decrease in background conductivity. With 1 mM of His, the highest resolutions and peak areas of GLYP, GLUF, and MPPA were obtained. Moreover, Figure 1(c) represents the result of varying the pH from 2.50 to 3.50 on the separation performance between GLYP, GLUF, and MPPA. When the pH of BGE decreased, the migration time and separation performance increased due to the increasing velocity, as mentioned. The best resolutions were achieved at the condition of pH 2.50, but at the same time, the peak heights were the worst for all analytes. Therefore, the optimum condition was selected with a pH of 2.75.

### 3.2. Development of the CE-C4D Methodology for Determination of AMPA

As mentioned, for analysis of AMPA (with pKa values of 1.8; 5.4; and 10) on the second channel, the selected BGE solution must have a pH higher than pK_a2_ to ensure that most of these analytes exist in anion form. A study by Hong et al. showed that the pH range of 5.8–6.8 was suitable for AMPA detection [40]. However, in that work, the used His/MES buffer needs to be added methanol at a concentration of 3% (v/v) to separate AMPA and GLUF. Therefore, BGEs composed of His/MOPS were used to provide the higher separation in this study. In addition, the separation of an anion at high pH is strongly affected by the electroosmotic flow (EOF). Consequently, CTAB was added to BGE solutions to reduce the hindrance of EOF. At the initial condition, the pH and CTAB concentration in buffer solution were fixed at 6.5 and 20 *μ*M, respectively.

**Figure 2:**
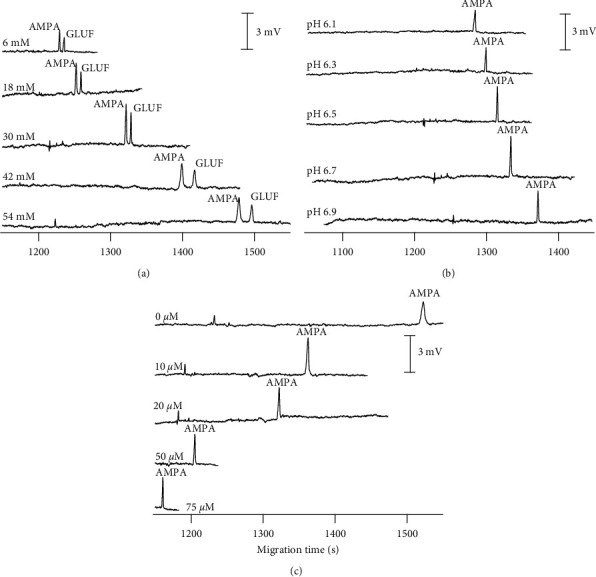
Electropherogram for the optimization of BGE compositions for determination of AMPA. CE conditions: voltage: 20 kV from the detection side; uncoated fused silica capillary with ID = 50 µm, Lt = 70 cm, and Leff = 62 cm.

The impact on AMPA detection performance of His amounts ranging from 6 to 54 mM was examined. As is shown in Figure 2(a), increasing the His portion resulted in a longer analysis time, and the best shape and highest signal of AMPA were obtained at the concentration of 30 mM. The pH of 30 mM His/MOPS buffers was then explored between 6 and 7, agreeing with previous studies. The results in Figure 2(b) indicated that a higher pH caused a longer migration time. This observation is attributable to the strength of the EOF, which has a more suppressive effect on AMPA migration at greater pH. In comparison, the decrease in MOPS concentration due to the drop in pH yielded a reduction in background conductivity but an enhancement in height peaks. Additionally, the pH 6.7 condition produced a better signal at an appropriate analysis period and was chosen for AMPA determination. Finally, the effect of CTAB concentration, the EOF modifier, was investigated in the range of 0–75 *µ*M. As illustrated in Figure 2(c), when there was no CTAB, the analysis time was extremely long (1500 seconds), owing to the EOF's influence. The migration times lowered, and the AMPA signals decreased as CTAB concentrations increased. As a result, a CTAB concentration of 10 *µ*M was chosen as the optimum condition.

### 3.3. Method Optimization for Solid-Phase Extraction

Due to their acid-base properties, GLYP, GLUF, AMPA, and MPPA could be dissociated into anions at the pH of tea infusions (5.30–6.03) [47], so it was suitable for using ion-exchange SPE columns, such as WAX columns. Therefore, in this study, the analytes were retained on the WAX cartridges as anions by ion-pair interaction with the sorbent of ammonium ion and eluted by an acidic eluent, HCl/MeOH. Two factors were explored for optimizing the SPE technique for sample enrichment: the HCl concentration and the eluent volume. The efficiencies of the SPE process were calculated as the ratio of the measured concentrations to the initial spiked concentration (10 µg/L).

**Figure 3:**
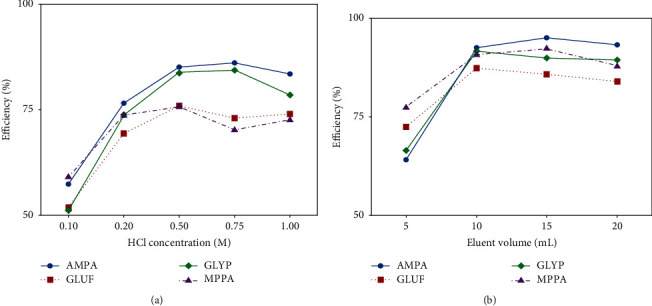
Electropherogram for the optimization of SPE procedures. CE conditions: voltage: 20 kV from the detection side; uncoated fused silica capillary with ID = 50 µm, Lt = 70 cm, and Leff = 62 cm.

First, the effect of HCl concentration was researched between 0.1 and 1 mM (see Figure 3(a)). It was discovered that raising the concentration of HCl to 0.5 M raised the extraction effectiveness of all target chemicals and then declined at higher concentrations. These findings substantiated the choice of the 0.5 M HCl/MeOH as optimum eluent. Subsequently, the HCl/MeOH volume was studied between 5 and 20 mL to determine the extraction efficiency. As shown in Figure 3(b), the extraction efficiency of the target compounds rose as the solvent volume increased from 5 to 10 mL and remained almost constant above 10 mL. As a result, the SPE operations were performed using a volume of 10 mL of 0.5 M HCl/MeOH. At the discovered optimal conditions, the SPE recoveries of GLYP, GLUF, AMPA, and MPPA were 91.5, 87.2, 92.5, and 92.7%, respectively, and the enrichment factors were 100 for all analytes.

### 3.4. Method Validation

Table 1 summarizes the salient performance data for the determinations of GLYP, GLUF, AMPA, and MPPA with the developed CE-C^4^D methodologies. The method detection limit (MDL) and method quantitative limit (MQL) for all target chemicals in the tea infusion sample were at the ppb level within the acceptable range of 0.54–5.19 µg/L. The comparison of results with other methods is shown in Table 2, and it can be observed that, though higher than in chromatographic methods, the MDL values found in this study were similar to those of previous studies using the CE technique. The linear ranges were from MQLs to 400, 200, 200, and 1,600 µg/L for GLYP, GLUF, MPPA, and AMPA, respectively. The calibration curve of each analyte was established in the range of 5.0–100 µg/L with good linearities of *R*^2^ > 0.998. According to regulation standards [48], the maximum allowable limit for GLYP in drinking water is 0.1 µg/L and in dry tea leaves is 1 mg/kg, which corresponds to 10 µg/L in tea infusion using the developed analytical procedure of brewing 1.0 g of tea leaves in 100 mL. Thus, the MDL and MQL for GLYP in this study indicated that although the developed analytical method could not meet the EU standard for drinking water, it met the objectives for detecting GLYP residues in tea leaves.

The intra-assay precision was assessed by comparing the relative standard derivation (RSD) values for peak area and migration time measurements (*n* = 11) taken within one day under optimum conditions and with a standard solution of 2 mg/L for each analyte. The interassay precision was measured using the same solution but over a seven-day period (*n* = 7). The recovery was determined using the standard addition method with the concentrations of 5, 10, and 20 µg/L spiked in a real matrix of the TD2 sample. The RSD values of migration time and peak area of each analyte were less than 7% and 9% in the intra- and interday precisions, respectively. The majority of obtained values for repeatability and reproducibility met the AOAC accuracy criterion [49]. The recoveries of four target compounds ranged between 80.6 and 99.6%, indicating that the procedure produced dependable results with high precisions.

### 3.5. Application of Analytical Procedures

**Figure 4:**
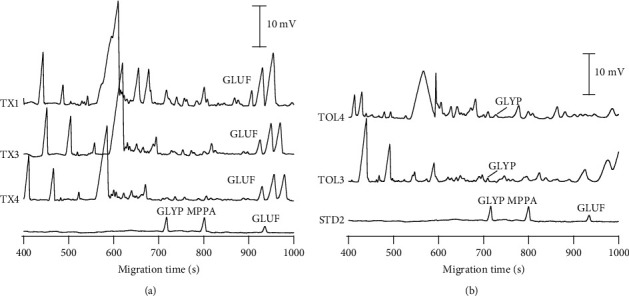
Electropherograms of the green (a) and oolong (b) tea infusion samples. Other CE conditions as in Figure 1 and Figure 2, respectively.

As can be seen in Table 3 and Figure 4, three tea infusions prepared from green teas (TX1, TX3, and TX4) were detected with GLUF in the range of 45.1–53.9 µg/L, while there were two samples of oolong tea infusions (TOL3 and TOL4) detected with GLYP at concentrations of 5.34–10.74 µg/L. AMPA and MPPA were not detected in all tea infusion samples. Although the CE-C^4^D methodology can be used to screen for GLYP or GLUF residues and control tea product quality, specific analytical techniques are usually required for further validation before such mandatory reporting is performed.

## 4. Conclusions

Two analytical procedures using a dual-channel CE-C^4^D system effectively developed a convenient and cost-effective approach for quantifying GLYP, GLUF, AMPA, and MPPA in tea infusions. The targeted analytes were extracted and concentrated by SPE and then were analyzed on two separate CE channels. The MDLs for GLYP, GLUF, AMPA, and MPPA were 0.80, 1.56, 0.56, and 0.54 *μ*g/l, respectively. GLYP was found in two infusion samples of oolong tea with concentrations ranging from 5.34 to 10.74 µg/L, and GLUF was recognized in three samples of green tea infusion in a total of 16 analyzed samples at concentrations in the range of 45.1–53.9 µg/L. The research has contributed to the development of CE-C^4^D applications in food and beverage quality control, and the developed method can be applied to local laboratories with modest budgets and limited expertise.

## Figures and Tables

**Table 1:** Salient performance data for the determinations of GLYP, GLUF, AMPA, and MPPA with the purpose-made CE-C4D system. Conditions: channel 1: GLYP, GLUF, and MPPA analysis: BGE solution: 1.0 M His/Ace (pH = 3.0); voltage: 20 kV; capillary: uncoated fused silica, 50 µm ID, *L*_t_ = 70 cm (*L*_eff_ = 49 cm). Channel 2: AMPA analysis: BGE solution: 30 mM His/MOPS (pH = 6.7); voltage: 20 kV; capillary: uncoated fused silica, 50 *µ*m ID, *L*_t_ = 70 cm (*L*_eff_ = 62 cm).

Analytes	MDL (*µ*g/L)	MQL (*µ*g/L)	Linear range (*µ*g/L)	Linearity (*R*^2^)	Intra-assay precision (*n* = 7)		Interassay precision (10 days)		Recovery (%)
					RSD % for PA	RSD % for MT	RSD % for PA	RSD % for MT	
GLYP	0.80	2.68	5.0–100	0,999	6.2	0.8	8.3	6.1	87.5–99.6
MPPA	0.56	1.87	5.0–100	0,998	4.6	0.7	8.9	5.8	83.5–88.4
GLUF	1.56	5.19	5.0–100	0,998	5.6	0.6	8.3	5.5	88.7–92.8
AMPA	0.54	1.82	5.0–100	0,998	3.8	4.2	7.7	6.2	80.6–86.6

**Table 2:** Comparison of different analytical methods applied for the determination of GLYP, GLUF, AMPA, and MPPA.

Analytical technique	Sample	Linear range (*μ*g/L)	MDL (*μ*g/L)	Enrichment factor	References
LLE—UHPLC-MS/MS	Groundwater and surface water	0.1–100 (GLYP)	0.2	-	[27]
		0.1–100 (GLUF)	0.01		
		0.1–100 (AMPA)	0.1		

One-step purification/extraction—UPLC-MS/MS	River water	0.04–0.8 (GLYP)	0.004	250	[50]
		0.04–0.8 (GLUF)	0.005		
		0.04–0.8 (AMPA)	0.004		
		0.04–0.8 (MPPA)	0.004		

SPE—IC-HESI-MS/MS	Surface water	0.01–2.0 (GLYP)	0.01	-	[31]
		0.01–2.0 (GLUF)	0.01		
		0.01–2.0 (AMPA)	0.01		
		0.01–2.0 (MPPA)	0.01		

D-SPE^a^—CE-MS	Baby food	2–1000 (GLYP)	0.5	60	[33]
		0.5–500 (GLUF)	0.1		
		1–1000 (AMPA)	0.4		
		1–1000 (MPPA)	0.2		

FESI^b^—CE-C^4^D	Tap water	0.2–169 (GLYP)	0.1	1002	[40]
		3.6–1810 (GLUF)	2.0	245	
		2.2–1110 (AMPA)	2.2	257	

SPE—CE-C^4^D	Tea infusion	2.69–400 (GLYP)	0.80	100	This study
		5.19–200 (GLUF)	1.56		
		1.82–1600 (AMPA)	0.54		
		1.87–200 (MPPA)	0.56		

**Table 3:** Contents of GLYP, GLUF, AMPA, and MPPA in tea infusions determined with CE-C4D and the reference methods.

ID	Sample type	Origin	GLYP (µg/L)	MPPA (µg/L)	GLUF (µg/L)	AMPA (µg/L)
TX1	Green tea	VN	-	-	45.1 ± 2.53	-
TX2	Green tea	VN	-	-	-	-
TX3	Green tea	VN	-	-	53.9 ± 3.02	-
TX4	Green tea	England	-	-	52.5 ± 2.94	-
TX5	Green tea	Russia	-	-	-	-
TD1	Black tea	VN	-	-	-	-
TD2	Black tea	VN	-	-	-	-
TD3	Black tea	Sri Lanka	-	-	-	-
TD4	Black tea	England	-	-	-	-
TD5	Black tea	Russia	-	-	-	-
TD6	Black tea	VN	-	-	-	-
TD7	Black tea	England	-	-	-	-
TOL1	Oolong tea	VN	-	-	-	-
TOL2	Oolong tea	VN	-	-	-	-
TOL3	Oolong tea	VN	5.34 ± 0.33	-	-	-
TOL4	Oolong tea	Taiwan	10.74 ± 0.67	-	-	-

## Data Availability

The data used to support the findings of this study are available from the corresponding author upon request.

## References

[1] Meftaul I. M., Venkateswarlu K., Dharmarajan R. (2020). Controversies over human health and ecological impacts of glyphosate: is it to be banned in modern agriculture?. *Environmental Pollution*.

[2] Ma X., Wang B., Li Z. (2022). Effects of glufosinate-ammonium on male reproductive health: focus on epigenome and transcriptome in mouse sperm. *Chemosphere*.

[3] Gandhi K., Khan S., Patrikar M. (2021). Exposure risk and environmental impacts of glyphosate: highlights on the toxicity of herbicide co-formulants. *Environmental Challenges*.

[4] Hsiao J.-T., Pan H.-Y., Kung C.-T., Cheng F.-J., Chuang P.-C. (2021). Assessment of glufosinate-containing herbicide exposure: a multi-center retrospective study. *The American Journal of Emergency Medicine*.

[5] Druart C., Millet M., Scheifler R., Delhomme O., de Vaufleury A. (2011). Glyphosate and glufosinate-based herbicides: fate in soil, transfer to, and effects on land snails. *Journal of Soils and Sediments*.

[6] Geng Y., Jiang L., Zhang D. (2021). Glyphosate, aminomethylphosphonic acid, and glufosinate ammonium in agricultural groundwater and surface water in China from 2017 to 2018: occurrence, main drivers, and environmental risk assessment. *The Science of the Total Environment*.

[7] Cederlund H. (2022). Environmental fate of glyphosate used on Swedish railways - results from environmental monitoring conducted between 2007-2010 and 2015-2019. *The Science of the Total Environment*.

[8] Yan B., Lei L., Chen X. (2022). Glyphosate and glufosinate-ammonium in aquaculture ponds and aquatic products: occurrence and health risk assessment. *Environmental Pollution*.

[9] Poiger T., Buerge I. J., Bächli A., Müller M. D., Balmer M. E. (2017). Occurrence of the herbicide glyphosate and its metabolite AMPA in surface waters in Switzerland determined with on-line solid phase extraction LC-MS/MS. *Environmental Science and Pollution Research*.

[10] Botten N., Wood L. J., Werner J. R. (2021). Glyphosate remains in forest plant tissues for a decade or more. *Forest Ecology and Management*.

[11] Smedbol É., Lucotte M., Maccario S. (2019). Glyphosate and aminomethylphosphonic acid content in glyphosate-resistant soybean leaves, stems, and roots and associated phytotoxicity following a single glyphosate-based herbicide application. *Journal of Agricultural and Food Chemistry*.

[12] Goscinny S., Unterluggauer H., Aldrian J., Hanot V., Masselter S. (2012). Determination of glyphosate and its metabolite AMPA (aminomethylphosphonic acid) in cereals after derivatization by isotope dilution and UPLC-MS/MS. *Food Analytical Methods*.

[13] Kolakowski B. M., Miller L., Murray A., Leclair A., Bietlot H., van de Riet J. M. (2020). Analysis of glyphosate residues in foods from the Canadian retail markets between 2015 and 2017. *Journal of Agricultural and Food Chemistry*.

[14] Lemke N., Murawski A., Schmied-Tobies M. I. H. (2021). Glyphosate and aminomethylphosphonic acid (AMPA) in urine of children and adolescents in Germany - human biomonitoring results of the German Environmental Survey 2014-2017 (GerES V). *Environment International*.

[15] Ferreira C., Duarte S. C., Costa E. (2021). Urine biomonitoring of glyphosate in children: exposure and risk assessment. *Environmental Research*.

[16] Lesseur C., Pathak K. V., Pirrotte P. (2022). Urinary glyphosate concentration in pregnant women in relation to length of gestation. *Environmental Research*.

[17] European Union (2020). *Directive (EU) 2020/2184 of the European Parliament and of the Council of 16 December 2020 on the Quality of Water Intended for Human Consumption*.

[18] Zhang Q, Li T, Wang Q, LeCompte J, Harkess R. L, Bi G (2020). Screening tea cultivars for novel climates: plant growth and leaf quality of camellia sinensis cultivars grown in Mississippi, United States. *Frontiers of Plant Science*.

[19] Wang Y., Gao W., Li Y. (2021). Establishment of a HPLC-MS/MS detection method for glyphosate, glufosinate-ammonium, and aminomethyl phosphoric acid in tea and its use for risk exposure assessment. *Journal of Agricultural and Food Chemistry*.

[20] Yusà V., Sanchís Y., Dualde P., Carbonell E., Coscollà C. (2021). Quick determination of Glyphosate and AMPA at sub µg/L in drinking water by direct injection into LC-MS/MS. *Talanta Open*.

[21] Jansons M., Pugajeva I., Bartkevics V., Karkee H. B. (2021). LC-MS/MS characterisation and determination of dansyl chloride derivatised glyphosate, aminomethylphosphonic acid (AMPA), and glufosinate in foods of plant and animal origin. *Journal of Chromatography B*.

[22] Ramirez C. E., Bellmund S., Gardinali P. R. (2014). A simple method for routine monitoring of glyphosate and its main metabolite in surface waters using lyophilization and LC-FLD + MS/MS. Case study: canals with influence on Biscayne National Park. *The Science of the Total Environment*.

[23] Bressán I. G., Llesuy S. F., Rodriguez C. (2021). Optimization and validation of a liquid chromatography-tandem mass spectrometry method for the determination of glyphosate in human urine after pre-column derivatization with 9-fluorenylmethoxycarbonyl chloride. *Journal of Chromatography B*.

[24] Nørskov N. P., Jensen S. K., Sørensen M. T. (2019). Robust and highly sensitive micro liquid chromatography–tandem mass spectrometry method for analyses of polar pesticides (glyphosate, aminomethylphosfonic acid, N-acetyl glyphosate and N-acetyl aminomethylphosfonic acid) in multiple biological matrices. *Journal of Chromatography A*.

[25] Ulrich J. C., Ferguson P. L. (2021). Development of a sensitive direct injection LC-MS/MS method for the detection of glyphosate and aminomethylphosphonic acid (AMPA) in hard waters. *Analytical and Bioanalytical Chemistry*.

[26] Guo H., Gao Y., Guo D. (2019). Sensitive, rapid and non-derivatized determination of glyphosate, glufosinate, bialaphos and metabolites in surface water by LC-MS/MS. *SN Applied Sciences*.

[27] Demonte L. D., Michlig N., Gaggiotti M., Adam C. G., Beldoménico H. R., Repetti M. R. (2018). Determination of glyphosate, AMPA and glufosinate in dairy farm water from Argentina using a simplified UHPLC-MS/MS method. *The Science of the Total Environment*.

[28] Martin-Reina J., Dahiri B., Carbonero-Aguilar P. (2021). Validation of a simple method for the determination of glyphosate and aminomethylphosphonic acid in human urine by UPLC-MS/MS. *Microchemical Journal*.

[29] Guo H., Wang H., Zheng J., Liu W., Zhong J., Zhao Q. (2018). Sensitive and rapid determination of glyphosate, glufosinate, bialaphos and metabolites by UPLC-MS/MS using a modified Quick Polar Pesticides Extraction method. *Forensic Science International*.

[30] Carretta L., Cardinali A., Marotta E., Zanin G., Masin R. (2019). A new rapid procedure for simultaneous determination of glyphosate and AMPA in water at sub *μ*g/L level. *Journal of Chromatography A*.

[31] Geerdink R. B., Hassing M., Ayarza N. (2020). Analysis of glyphosate, AMPA, Glufosinate and MPPA with ION chromatography tandem mass spectrometry using A membrane suppressor in the ammonium form application to surface water of low to moderate salinity. *Analytica Chimica Acta*.

[32] Tůma P, Opekar F, Dlouhý P (2022). Capillary and microchip electrophoresis with contactless conductivity detection for analysis of foodstuffs and beverages. *Food Chemistry*.

[33] Liu J., Feng W., Tian M., Hu L., Qu Q., Yang L. (2021). Titanium dioxide-coated core-shell silica microspheres-based solid-phase extraction combined with sheathless capillary electrophoresis-mass spectrometry for analysis of glyphosate, glufosinate and their metabolites in baby foods. *Journal of Chromatography A*.

[34] Goodwin L., Startin J. R., Keely B. J., Goodall D. M. (2003). Analysis of glyphosate and glufosinate by capillary electrophoresis–mass spectrometry utilising a sheathless microelectrospray interface. *Journal of Chromatography A*.

[35] Chang S. Y, Liao C. H (2002). Analysis of glyphosate, glufosinate and aminomethylphosphonic acid by capillary electrophoresis with indirect fluorescence detection. *Journal of Chromatography A*.

[36] Wei X., Gao X., Zhao L. (2013). Fast and interference-free determination of glyphosate and glufosinate residues through electrophoresis in disposable microfluidic chips. *Journal of Chromatography A*.

[37] Chang S. Y., Wei M.-Y. (2005). Simultaneous determination of glyphosate, glufosinate, and aminomethylphosphonic acid by capillary electrophoresis after 9-fluorenylmethyl chloroformate derivatization. *Journal of the Chinese Chemical Society*.

[38] Molina M., Silva M. (2002). In-capillary derivatization and analysis of amino acids, amino phosphonic acid-herbicides and biogenic amines by capillary electrophoresis with laser-induced fluorescence detection. *Electrophoresis*.

[39] See H. H., Hauser P. C., Sanagi M. M., Ibrahim W. A. W. (2010). Dynamic supported liquid membrane tip extraction of glyphosate and aminomethylphosphonic acid followed by capillary electrophoresis with contactless conductivity detection. *Journal of Chromatography A*.

[40] See H. H., Hauser P. C., Ibrahim W. A. W., Sanagi M. M. (2010). Rapid and direct determination of glyphosate, glufosinate, and aminophosphonic acid by online preconcentration CE with contactless conductivity detection. *Electrophoresis*.

[41] Graf H. G., Biebl S. M., Müller L., Breitenstein C., Huhn C. (2021). Capillary electrophoresis applied for the determination of acidity constants and limiting electrophoretic mobilities of ionizable herbicides including glyphosate and its metabolites and for their simultaneous separation. *Journal of Separation Science*.

[42] Nguyen T. D., Vu M. T., Nguyen M. H., Duong H. A., Mai T. D., Pham H. V. (2021). A rapid and simple dual-channeled capillary electrophoresis with contactless conductivity detection method for the determination of adenosine, cordycepin, and inosine in ophiocordyceps sinensis-based products. *Food Analytical Methods*.

[43] Duong H. A., Vu M. T., Nguyen T. D., Nguyen M. H., Mai T. D. (2020). Determination of 10-hydroxy-2-decenoic acid and free amino acids in royal jelly supplements with purpose-made capillary electrophoresis coupled with contactless conductivity detection. *Journal of Food Composition and Analysis*.

[44] Nguyen T. D., Nguyen M. H., Vu M. T., Duong H. A., Pham H. V., Mai T. D. (2019). Dual-channeled capillary electrophoresis coupled with contactless conductivity detection for rapid determination of choline and taurine in energy drinks and dietary supplements. *Talanta*.

[45] Phan Thi L.-A., Ngoc N. T., Quynh N. T. (2020). Polycyclic aromatic hydrocarbons (PAHs) in dry tea leaves and tea infusions in Vietnam: contamination levels and dietary risk assessment. *Environmental Geochemistry and Health*.

[46] Mai T. D., Pham T. T. T., Pham H. V., Sáiz J., Ruiz C. G., Hauser P. C. (2013). Portable capillary electrophoresis instrument with automated injector and contactless conductivity detection. *Analytical Chemistry*.

[47] Bobkova A., Demianova A., Belej L. (2021). Detection of changes in total antioxidant capacity, the content of polyphenols, caffeine, and heavy metals of teas in relation to their origin and fermentation. *Foods*.

[48] Directive C. (1991). Council Directive 91/414/EEC of 15 July 1991 concerning the placing of plant protection products on the market. *Official Journal of the European Communities - Legislation*.

[49] Horwitz W. (2002). *AOAC Guidelines for Single Laboratory Validation of Chemical Methods for Dietary Supplements and Botanicals*.

[50] Vu C. T., Le P. T., Chu D. B. (2021). One-step purification/extraction method to access glyphosate, glufosinate, and their metabolites in natural waters. *Journal of Chromatography A*.

